# Proliferation of axial parenchymatic xylem cells is a key step in wound closure of girdled stems in *Pinus canariensis*

**DOI:** 10.1186/s12870-015-0447-z

**Published:** 2015-02-27

**Authors:** Víctor Chano, Rosana López, Pilar Pita, Carmen Collada, Álvaro Soto

**Affiliations:** GENFOR, Grupo de Investigación en Genética y Fisiología Forestal. ETSI Montes, Universidad Politécnica de Madrid, Ciudad Universitaria s/n, 28040 Madrid, Spain; Unidad Mixta de Genómica y Ecofisiología Forestal, INIA/UPM, Madrid, Spain

**Keywords:** Wound closure, Vascular cambium, Parenchymatic xylem cells, Conifers

## Abstract

**Background:**

Wounds caused by fire, herbivorism, rock impacts, etc. cause the direct loss of photosynthetic, storage and/or vascular tissue. In addition, they may entail other damages, such as desiccation of the exposed internal parts, or become a gateway to infection by fungi and other pathogens. To successfully overcome such injuries, plants must reorganize their meristems or even differentiate new ones, producing new traumatic tissues to cover the wound and restore the vascular connection.

**Results:**

In this work we analyse the anatomical growth response in conifers after debarking and injuring the vascular cambium, using *Pinus canariensis* as model species, due to its high wound recovery ability. Conversely to angiosperm woody species, this process is initiated and largely driven by the damaged vascular cambium and not by proliferation in the wound surface. We have detected alterations and switches in the divisions of cambial cells, associated to their position relative to the surface and edges of the wound, resulting in disordered traumatic xylem. We also describe the formation of column-like structures, after girdling, which are in part formed by the proliferation of xylem parenchymatous cells, associated to axial resin ducts.

**Conclusions:**

Abundant resinosis on the wound surface, typical of conifers, is an efficient barrier against opportunistic fungi, insects, etc. but it also hinders the healing process directly from the surface. Thus, wound closure must be largely carried out from the wound margins, being a much slower process, which very often remains unconcluded for long years. This work also describes for the first time the proliferation of inner parenchymatous cells to form column-like structures, which accelerates wound closure in girdled *P. canariensis.* Irregularities in the surface of the healing edge or column-like structures result in the production of disordered vascular tissues, compromising their future functionality, and which must be overcome through the fast restoration of the proper polarity in vascular cambium.

## Background

Throughout their usually long lives, trees can be affected by traumatic injuries caused by different agents, from herbivorism to forest fires, from avalanches in mountain environments to impacts from rocks and other material carried by floods or even from pyroclasts propelled by volcanic eruptions. In addition to the direct loss of photosynthetic and vascular tissues, these events can ease the entry and spread of fungi or other pathogens in the plant. The wound triggers a set of anatomical and physiological responses which avoid or hamper the possible expansion of the infection. Shigo [1,2] proposed the CODIT (Compartmentalization Of Decay In Trees) model to depict the response to wound displayed in secondary xylem. This model describes a series of radial, transverse and tangential walls in the xylem that ultimately confine the putative pathogen and its damages, resisting their spread. According to CODIT, chemical barriers are first developed in tissues existing prior to injury (constituting the so-called reaction zone), whereas further barrier is constituted by the newly formed tissues (barrier zone), which close the wound, leaving a more or less extensive scar in the xylem.

Many of the studies on the traumatic response have used angiosperm model species, especially those focusing in the molecular aspects of wound closure and regeneration (f. i. [3-5]), its hormonal control (f. i. [6,7]) or the anatomical process (f. i. [8-11]). Regarding this latter issue, different works describe callus and woundwood (new xylem contributing to wound closure) formation directly from the surface of a wound in the stem of angiosperm adult tress. For instance, Stobbe *et al.* [8], after removing a rectangular portion of bark, phloem and cambium in *Tilia*, report the formation of a disordered callus from the proliferation of immature xylem cells as a first protective layer, and the differentiation of a new vascular cambium within this callus. A similar process is described by Pang *et al.* [9] after completely debarking the trunk of *Eucommia ulmoides*. On its side, Zhang *et al.* [11] report that the protective callus is produced by the proliferation of ray cells in *Populus tomentosa*, followed also by differentiation of a new traumatic vascular cambium within the callus. However, the contrasting anatomical characteristics of gymnosperm and angiosperm xylem may underlie different healing processes leading to the lower regenerating capability of the former.

In gymnosperms, most of the works in this area have focused in the formation of resin ducts in conifers in response to mechanical or insect-mediated injuries and fungal infection, particularly from a molecular point of view ([12-14], in *Picea*), or in the effect on wood growth patterns (early-late wood ratio, ring width, formation of traumatic resin ducts…) ([15,16] in *Picea*; [17] in *Picea* and *Larix*; [18,19] in *Picea, Abies* and *Larix;* [20,21] in *Pinus pinaster*; [22] in *Pseudotsuga, Larix* and *Pinus ponderosa*). Very recently, Stoffel and Klinkmüller [23] applied 3D X-ray computed tomography to analyze the long-term effects of wounding on xylem in *Abies alba, Larix decidua* and *Picea abies*. Conversely, very few studies have addressed the wound closure process itself from an anatomical point of view in conifers. Especially noteworthy are the paramount works of Mullick [24], Oven & Torelli [25,26] or Wahlström and Johansson [27], in different conifers.

Most of these works have focused on alpine species, which are often damaged by rockfall impacts, while very few works have focused on species with higher regeneration capacity, such as those adapted to volcanic environments [28]. In this work we analyze the anatomical healing in *Pinus canariensis.* This pine, with a comparatively abundant xylem parenchyma, is a suitable model species to study wound response in conifers, since it shows an extraordinary healing and even resprouting ability, highly uncommon among gymnosperms, particularly in the adult stage [29,30]. These features could be linked to *P. canariensis* evolutionary history, driven by the successive volcanic eruptions and subsequent re-colonizations in the Canary Islands [31]. We have used younger plants than previous works, analyzing the response not only in the xylem or phloem but also in the cortical parenchyma, and have performed both fenestration wounds and complete girdling.

From another point of view, several works in the last decades have paid attention to the establishment of polarity and organization of tissues in the developing embryo and apical meristems and differentiation of primary vascular tissues focusing on the balance and hormonal signals that determine these processes (f.i. [32-34]). However, little is still known about the reorganization of traumatic tissue, mainly when it affects the lateral meristem. In this work we focus on the anatomical aspects of this reorganization. Additionally, we describe for the first time the formation of column-like structures, as essential elements of the wound closure process after girdling.

## Material and methods

### Plant material and mechanical wounding

Three years old Canary Island pines grown in nursery at UPM facilities were used for this study. Twenty four trees were grown in 3:1 (v/v) peat:vermiculite, in 650 ml cone-containers first and 5 liters containers after the first year. At the moment of this study trees were approximately 150 cm high, with a diameter of 2–3 cm.

**Figure 1:**
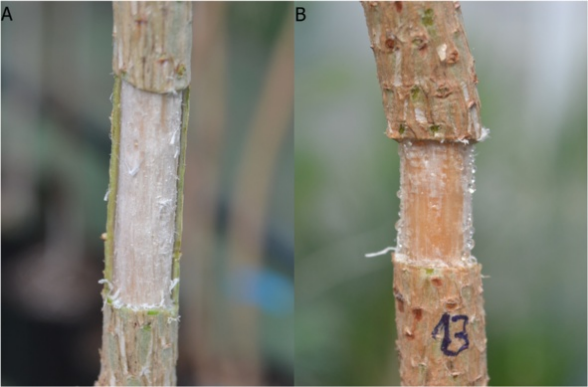
**Mechanical wounds. A**: Fenestration wound, removing bark, phloem and vascular cambium from a rectangular window 4 cm high and spanning half the circumference of the stem. **B**: Girdled stem 2 cm high. Abundant resinosis in wound surface is clearly visible.

Two kinds of mechanical wounds were performed on the stem of pines with a scalpel (twelve trees per treatment). We performed fenestration wounds in 12 trees, removing bark, phloem and vascular cambium from a rectangular window 4 cm high and spanning half the circumference (Figure 1A). Another set of 12 trees were completely girdled, and bark, phloem and vascular cambium were removed from a 2 cm high ring (Figure 1B).

### Bright-field and UV microscopy

In the laboratory, three samples of fenestrated stem were collected for microscopy analysis at four dates during the healing process, based on macroscopic observation: 8, 15, 28 and 50 days after wounding. As well, two stems were collected and processed at 10, 40, 60, 100 and 150 days after girdling. All samples were cut with a Leica SM2400 microtome the same day they were collected. For bright-field microscopy, cross and longitudinal sections (20–30 μm thick) were treated with sodium hypochlorite, washed with distilled water and then stained for 2 min with 1% safranine (w/v) and 1 min with 1% alcian blue (w/v), washed with distilled water, and dehydrated with ethanol series, based on Heijari *et al.* [35].

Additional 20–30 μm thick cross sections of wounded stem were stained for tannins, callose and suberin observation, using a fluorescence microscope (excitation at 340–380 nm, and 410–450 nm barrier filters) (Olympus BX51). The phloroglucinol-HCl test [36] was performed for visualization of lignified and suberized cell walls under tungsten and UV light. We first poured a drop of 1% phloroglucinol:ethanol solution (w/v), and then added a drop of 35% HCl. While lignin appears stained in red under white light, quenching of lignin autofluorescence under UV light by phloroglucinol-HCl allows the identification of suberized tissues [37,38]. For tannins detection, sections were stained with a drop of vanillin alcohol saturated, following by adding a drop of HCl 35%, based in Vanillin-HCl test performed by Gardner [39]. For callose detection, sections were stained with 1:1 (v/v) mix of 0.005% anilin blue (w/v) and 0.15 M K_3_PO_4_ pH 8.2, based on Currier & Strugger [40].

## Results and discussion

As occurs in most conifers, the first response to wounding in *P. canariensis* is an abundant resinosis in the wound surface, but the wound closure process takes place mostly from the wound edges. Certain differences have been detected between healing from the upper and from the side margins.

### Wound closure from lateral edges

When the tree suffers fenestration wounding, and the stem is not completely girdled, most of the wound closure takes place from the lateral wound edges. Different steps can be distinguished in the process:

1. Lignification and suberization of cortical parenchymatous cells. The first observable response was detected in the cortex, 8 days after wounding. Approximately 2–8 cells behind the lateral edge of the wound, a 3–5 cells wide line of parenchymatous cells got lignified (Figure 2A), providing a first barrier to minimize water loss and the possible entrance of opportunistic pathogens in the cortex, as first described by Mullick [24] for injuries in the bark of fir, hemlock and thuja. Seven days later, suberin is also detected (Figure 2B-C). This time of response is similar to the ones reported for other conifers [27,41]. However, while for young twigs of *Picea abies, Thuja orientalis* or *Metasequoia glyptostroboides* just a tenuous lignification can be observed in the injury boundary seven-ten days after wounding [39], a layer of strongly suberized cortical parenchymatous cells is already detectable by that time in *P. canariensis*.

**Figure 2:**
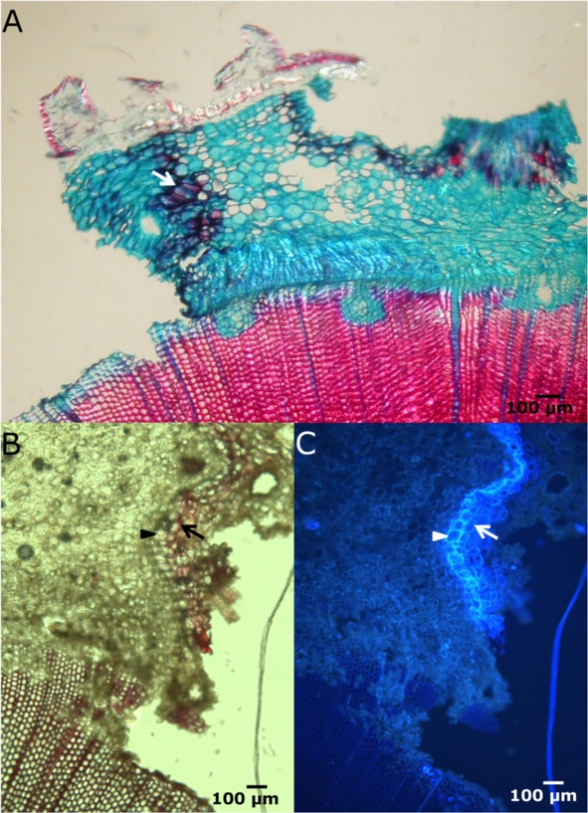
**Lignification and suberization of cortical parenchymatous cells. A**: Cross section of the lateral margin of a fenestration wound, 8 days after wounding, stained with safranine and alcian blue and seen in bright-field microscopy. Cortical parenchymatous cells in the border got lignified (arrow). **B-C**: Lateral edge of the wound seen by bright-field **(B)** and fluorescence microscopy **(C)**, stained with phloroglucinol-HCl. Protective barrier of lignified (arrow) and suberized (arrowhead) parenchymatous cells in the cortex. Reddish staining under white light reveals the presence of lignin, while higher fluorescence intensity under UV light corresponds to suberin deposits in cell walls.
2. Development of traumatic periderm in the cortex. Two to four weeks after wounding a traumatic phellogen differentiates just behind the first lignified boundary and starts to divide (Figure 3). This traumatic periderm contacts with the original periderm and the llignified and suberized cells of the callus (see below), forming a continuous impervious barrier. The cells outside this phellem dry out and die, isolating the pathogens that could have infected the exposed cortical cells and blocking the infection.

**Figure 3:**
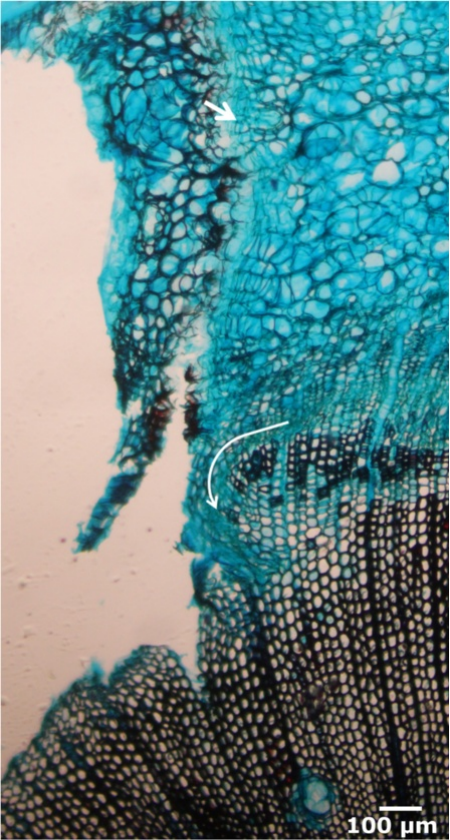
**New traumatic periderm and initiation of healing callus.** Cross section in bright-field microscopy of the lateral edge 15 days after wounding. New traumatic periderm (arrow) in the cortex. Proliferation in the cambial zone, curving the cambium inwards (white curved line).
3. Initiation of a healing callus. Approximately at the same time as the formation of the traumatic periderm within the cortical parenchyma, initial proliferation in the cambial zone, close to the lateral edge of the wound, is also perceptible (Figure 3). The cambium twists inwards, heading the surface of the wound, probably due to a very high number of multiplicative, radial anticlinal divisions, which generate additional cambial cells, as discussed by Zajaczkowska in *P. sylvestris* [42]. The proportion of radial anticlinal divisions is related negatively with the distance to the healing border, i. e., they are more frequent near the border, and ultimately would lead to the reconstruction of the cambial circumference. Simultaneously, first periclinal divisions give rise to parenchymatous cells outwards, which form a protecting callus. As occurs with the first response in the cortical parenchyma, the outer part of this callus gets lignified and suberized. Soon after, a new traumatic phellogen differentiates in the outer part of the parenchymatous healing edge, developing a new periderm (Figure 4A-B). As reported by Oven & Torelli [26], no periderm is formed in the ventral part of the healing callus. In the wound surface, several tracheids are filled with tannins (Figure 4C-D).

**Figure 4:**
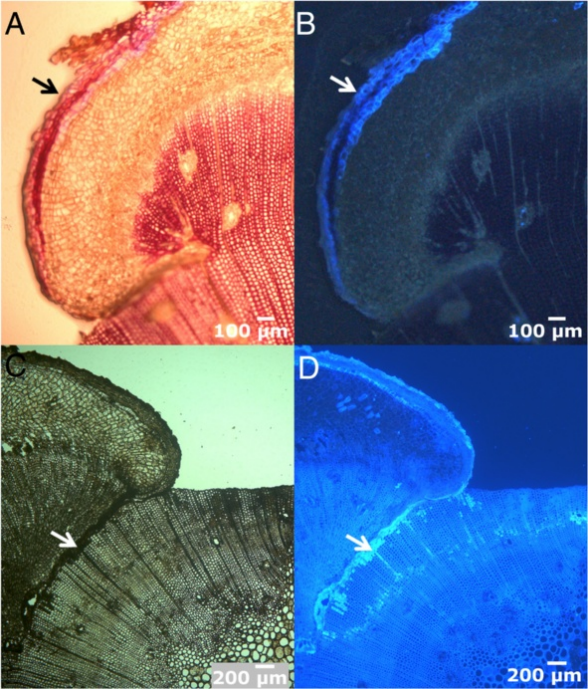
**Progress of the healing callus.** Cross sections of the lateral edge 28 days after wounding, in bright-field **(A** and **C)** and fluorescence microscopy **(B** and **D)**, showing the new suberized periderm **(A, B**, arrows; stained with phlorogucinol-HCl). Close to the ventral part of the healing tissue, several xylem cells appear filled with tannins **(C**, **D**, arrow; stained with vanillin-HCl).
4. Differentiation of vascular tissues. As the end of the cambium spreads further away towards the centre of the wound due to radial anticlinal divisions, new vascular tissues are generated by additive periclinal divisions of cambial cells. Xylem development via centripetal divisions forces the cambium to recover its normal position, parallel to the organ surface (Figure 5). This first traumatic xylem shows a high proportion of resin ducts, axial parenchyma, and irregular shaped tracheids, as already described for other species (e.g. [22,42,26]). On its side, phloem starts to differentiate later than xylem

**Figure 5:**
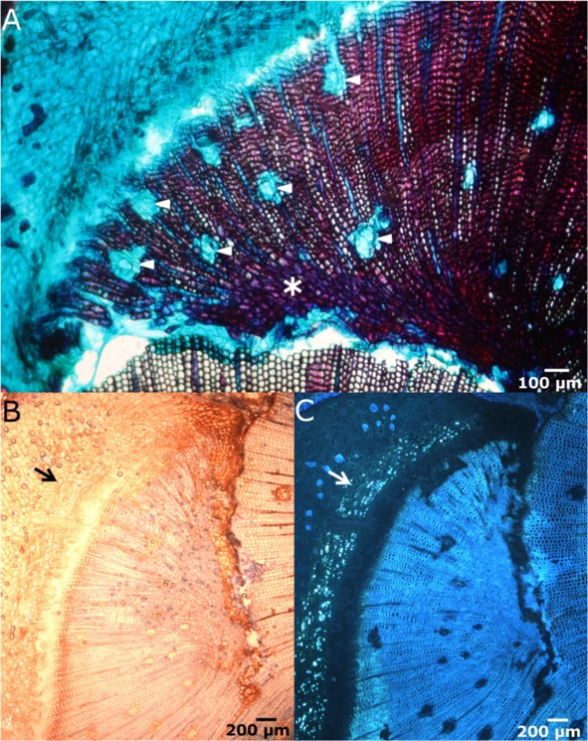
**Differentiation of vascular tissues. A**: Cross section in bright-field microscopy of the lateral edge 50 days after wounding. Healing vascular tissues show a high proportion of resin ducts (arrowheads), axial parenchyma and irregular shaped tracheids (asterisk). **B-C**: Cross sections after staining with aniline blue for callose detection in bright-field **(B)** and fluorescence microscopy **(C)** showing the presence of differentiated secondary phloem (arrow).
5. Wound closure. The stages 3 and 4 may continue for several growing seasons (depending on the size of wound and vigour of the tree), making the lateral healing edges to grow over the wound surface, until they finally get in contact and merge. As described by Hamada *et al.* [10], a high proportion of parenchymatous cells is appreciable in the ventral part of the traumatic xylem. The cells of the thin traumatic periderm and the parenchymatous callus cells in the edges collapse as cambium gets closed again and wood formation progresses in this area (Figure 6).

**Figure 6:**
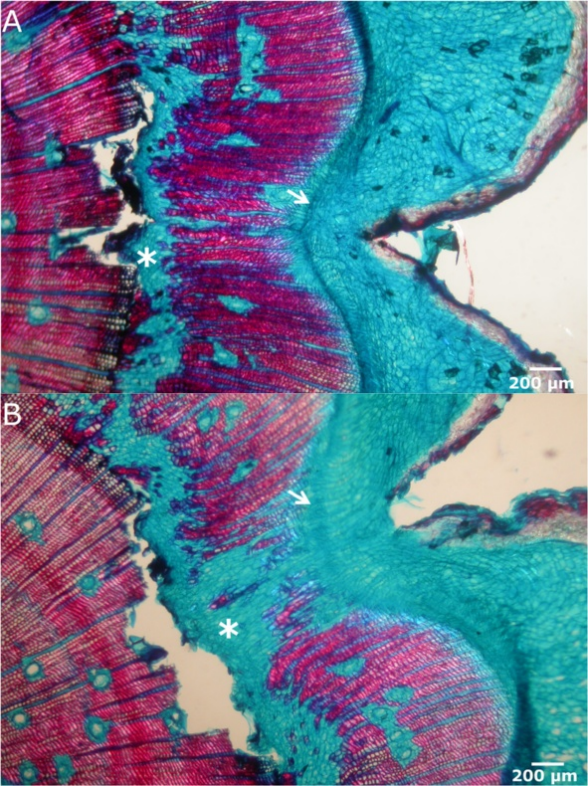
**Wound closure.** Microscopic view in bright-field microscopy of cross sections of a recently closed wound. Both lateral edges have met and the vascular cambium circumference is closed (arrows). A high proportion of parenchymatous cells in the ventral part of traumatic xylem is appreciable (asterisks).

### Wound closure from the upper margin

When the stem is completely girdled and there is no lateral edge left, healing is expected to be accomplished from the upper and lower edges of the wound. These injuries are usually much more dangerous and difficult for the tree to overcome, since phloem sap flow is entirely interrupted by the wound. Most trees cannot survive such damage, even among angiosperms.

**Figure 7:**
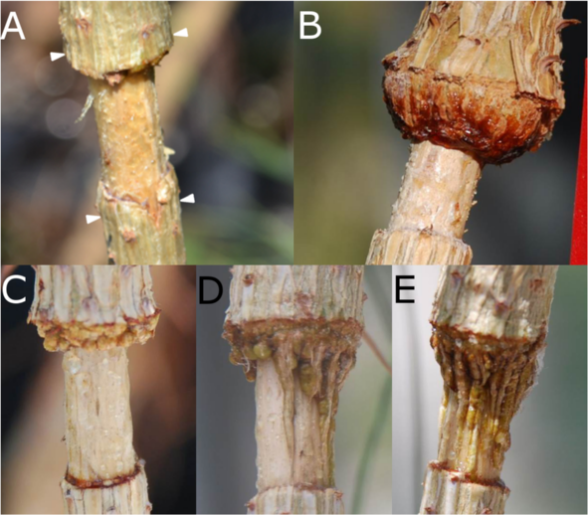
**Macroscopic view of the healing process in girdled stems. A**: Increase of stem diameter above the wound 10 days after girdling. **B**: First growth of parenchymatous tissue causes a bulge in the upper edge of the wound (40 days after girdling). **C**: Lumpy appearance 60 days after girdling, caused by the development of numerous protuberances in the upper edge. **D**: Column-like structures developed from the protuberances in the upper edge and axial parenchyma (see text for details) (100 days after girdling). **E**: Column-like structures reaching the lower margin and restoring the vascular connection (150 days after girdling).

**Figure 8:**
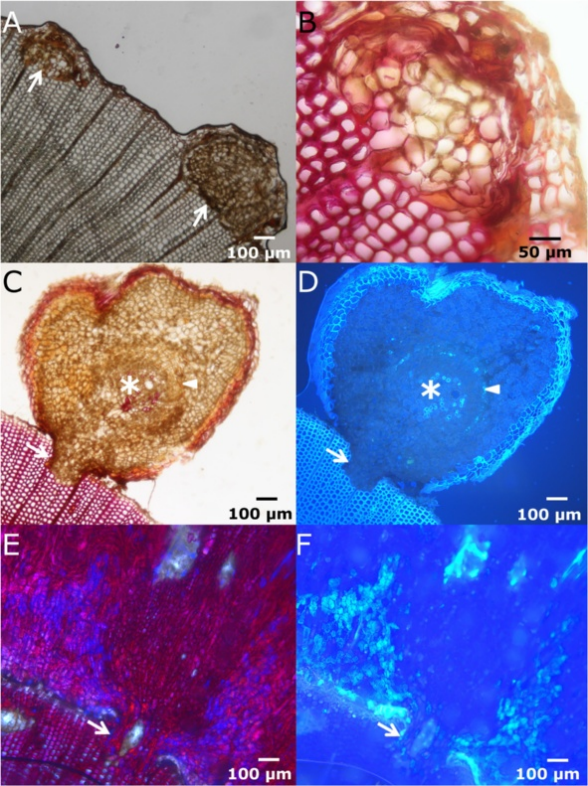
**Microscopic view of the column-like structures. A-B**: Early steps of development of column-like structures, in bright-field microscopy. **C-D**: Cross section of a column-like structure in bright-field **(C)** and fluorescence microscopy **(D)** showing differentiation of vascular tissues in a fan-shapped disposition. At this stage, few cells are completely lignified (asterisk). Arrowhead in D indicates the position of phloem, stained with aniline blue. The arrows show the union zone with the pre-wound xylem. **E-F**: Cross sections in bright-field and fluorescence microscopy, respectively, showing a column-like structure embedded in the healing tissue. The arrow in both pictures indicates the union zone.

Conversely, after girdling, *Pinus canariensis* displays an active growth from the upper edge, being often able to reconnect the phloem and surmount the injury if the removed ring is not too wide. The sequence of tissue differentiation in the upper edge is similar to the one described for the lateral edges. However, this downwards process shows some differences, with easily recognizable steps (Figure 7):

1. Swelling of the upper section. Immediately after wounding sieve cells are sealed, hampering the loss of sap. The resulting sap accumulation leads to a conspicuous bulge in the upper part of the wound (Figure 7A, 10 days after girdling), as described by Singh *et al.* [43] or de Schepper *et al.* [44].
2. First traumatic growth. Shortly after that, a growing border analogous to the one produced in the lateral edges of fenestration wounds is formed in the upper edge (Figure 7B, 40 days after girdling). Conversely, no traumatic growth is detected in the lower margin, and even a slight reduction in diameter due to desiccation of the first layers of exposed cells can be observed, which can be related to the profuse resprouting induced below the injury in *P. canariensis*. In fenestration wounds, growth from the upper margin does not go further this step and final wound closure must be achieved from lateral edges, as described above.
3. Protuberances. Later on, the upper growing edge acquires a lumpy appearance, with the development of numerous protuberances in it (Figure 7C, 60 days after girdling).
4. Column-like structures. Those protuberances develop downwards in column-like structures, which eventually achieve the lower edge of the wound, restoring the vascular connection (Figure 7D-E, 100 and 150 days after girdling, respectively). These structures do not only develop through the basipetal growth of the protuberances in the upper edge. Instead, we have observed that they also originate 5–6 cells below the wound surface, by means of the proliferation of parenchymatous cells surrounding constitutive axial resin ducts (Figure 8). We observed a first proliferation of parenchymatous cells and an early development of a periderm in the outer face of this structure (Figure 8A-B). The growth of this structure makes it break through the remaining tracheids to the wound surface. Subsequently, a column of vascular tissues differentiates, which can be observed with an approximately semicircular shape in a cross section, with phloem surrounding the outer face of xylem (Figure 8C-D). Subsequent growth of woundwood from the upper edge finally engulfs these structures (Figure 8E-F). Interestingly, these structures have only been detected after girdling, and not in fenestration wounds. This observation is consistent with a hormonal control of the regeneration process. Thus, it is well known that a high cytokinin/auxin ratio can lead to the development of shoots from a callus, while the opposite can induce roots [45]. In this case, the interruption of phloematic sap flow causes a noticeable increase of auxin in the upper margin on the wound and alters the cytokinins flow from the roots [46], which can underlie the formation of protuberances and column-like structures.

Contrarily to Oven & Torelli [26] in mature trees of other conifer species, we have not detected perceptible proliferation from phloem cells, neither in fenestration nor in girdling wounds. In the same way, we have not detected hyperplasia and proliferation from radial cells in the wound surface, as reported in *Populus tomentosa* [11]. Notwithstanding, radial parenchymatous cells keep their proliferating capability in pines. Thus, Kuroda and Shimaji [47] described how after sticking a needle deep in the xylem and removing it, an axially oriented bubble is formed within the xylem, not exposed to open air; subsequently, affected radial parenchymatous cells proliferate, filling the bubble and, finally, forming a resin pocket. On the contrary, open wounds as the ones made here, debarking the stem, break preexisting axial and radial canals, whose resin covers immediately the wound surface, preventing the entry of pathogens, but hindering further proliferation from immature xylem cells and radial parenchyma in this area, as occurs in angiosperms.

Although Ballesteros *et al.* [20] report that *“Pinus* do not normally form traumatic ducts and individual canals appear dispersed and only rarely in tangential bands”, there is a noticeable increase in the formation of axial parenchyma and resin ducts in tangential rows in the healing tissues and surrounding the wound (appreciable in Figures 4 and 5), especially above it, as already reported for other conifers, as *Pinus nigra* [48], *Picea abies* [15,49], *Larix decidua* [50,49] or *Cedrus libani* [51]. Actually, one of the main induced direct defenses in conifers after mechanical wounding, herbivore damage or fungal elicitation is the formation of traumatic resin ducts in the xylem, arranged in tangential rows [52] and this is the basis of resin exploitation, an important industry in the past, superseded in the 20^th^ century by the use of petroleum derivatives, but with increasing interest in the last years.

### Xylem and phloem differentiation

A noticeable feature, differing from other species, is the delayed differentiation of traumatic phloem. While in angiosperms phloem reconnection is achieved shortly after wounding through the differentiation of phloem elements within the parenchymatous callus (as in *Populus tomentosa*, [11]) or even by transdifferentiation of immature xylem elements (as in *Eucommia ulmoides*, [9]), before the development of a new traumatic vascular cambium, we have observed that in *P. canariensis* wound phloem starts to differentiate only after xylem. After severe stem wounding, the plant still needs to supply with nutrients the living tissues below the injury for survival. Additionally, as reviewed by Clarke *et al.* [53], one of the main factors enabling resprouting after trauma (a common response in angiosperms, but rare among gymnosperms, being *P. canariensis* a remarkable exception) is an efficient resourcing of a viable bud bank. In the same way, healing tissues also constitute an important resource sink. In fenestration wounds this supply could be accomplished by the remaining phloem on both sides of the injury. Nevertheless, in natural conditions *P. canariensis* endures large injuries, usually together with intense defoliations, as the ones caused by volcanic eruptions, so that very often foliage cannot provide enough nutrients to healing or resprouting tissues or to the root and stem below the injuries. In these cases, it is very likely that the reserves stored in the comparatively abundant radial and axial parenchyma [54] are used to provide these tissues with the required nutrients. However, if the damage is too intense or if the reserves are starved by previous, recent stresses, regeneration ability is reduced and the tree ultimately cannot heal the wound.

### Orientation of healing tissues

**Figure 9:**
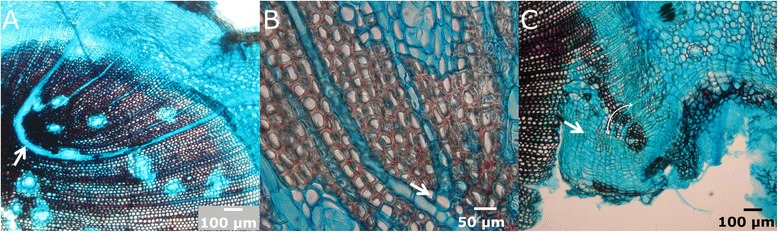
**Abnormal orientation in healing tissues. A-B**: Cross sections of lateral edges. The arrows indicate U- and Y-shaped rays, respectively. **C**: Cross section of a lateral edge few days after wounding. The white arrow indicates the thick parenchymatous callus developed inwards. The white curve line marks a U-shaped cell alignment.

Cambial cells must perceive somehow their position relative to the surface of the organ. Thus, additive divisions, which yield new xylem and phloem elements, usually take place according to a plane parallel to the surface of the organ, and they are also known as periclinal divisions. On the other side, multiplicative divisions, giving raise to new cambial cells, occur according to an axial anticlinal plane, perpendicular to the surface. Our results suggest that the position of the surface closest to the cambial zone determines the direction of periclinal and radial anticlinal divisions. Thus, close to the end of the wounded, open cambium, periclinal divisions go parallel to the wound lateral edge and perpendicular to the wound surface, and to normal, non-traumatic periclinal divisions. Nevertheless, due to the curvature of the cambium there can be a zone where the closest organ surface is detected in two different directions. In this case, sometimes a switch in the polarity of orientation of periclinal and radial anticlinal divisions takes place, leading to abnormal U- and Y-shaped arrays of cells coming from the same cambial initial. (Figure 9A-B).

Many works report the incidence of the alteration of hormone flux caused by wounds in the orientation, directionality of cell division and subsequent disorganization of wound xylem (e.g., auxins are involved in the specification of polarity in primary meristems, as reviewed by Berleth & Sachs [32]; ethylene production is induced by mechanical stress, as reported by Telewski & Jaffe [55], in *P. taeda*). However, mechanoperception also determines the directionality of divisions, as shown in thigmomorphogenesis studies and reviewed by Telewski [56]. The eminent work of Brown & Sax [57] shows that mechanoperception of the pressure exerted by surrounding cells determines the differentiation of phloem and xylem. Our results support the involvement of mechanoperception in the alteration of the normal pattern of cambium additive divisions, probably concomitantly with hormone flux and maybe even other factors, such as, for instance, the incidence of light.

If the injury penetrates in the xylem, parallel to the cambium, a similar switch in the direction of divisions can take place, and the very first multiplicative division can occur inwards; further periclinal additive divisions producing xylem will separate cambial cells, giving rise to U-shaped cell alignments in the xylem and forcing the cambium to acquire a “hairpin” shape (Figure 9C). The inner part of the “cambial hairpin” undergoes additive divisions inwards, producing comparatively large cells, with thin primary cellulosic walls, consistently with the results of Brown & Sax [57] for *P. strobus*. In that work, after partially removing a longitudinal strip of bark, keeping it attached at the acropetal end, the vascular cambium in the inner face of the strip produced a parenchymatous callus inwards. In our case, these divisions are very profuse, resulting in a thick callus advancing from the side edges to the center of the wound surface. These cells, coming from the vascular cambium, are not as disordered as the callus formed in angiosperm, which comes from the proliferation of radial parenchyma or immature xylem elements [8,9,11]. On the contrary, these cells in *P. canariensis* appear aligned perpendicularly to the surface, as corresponds to the result of periclinal divisions of the cambium (Figure 9C).

**Figure 10:**
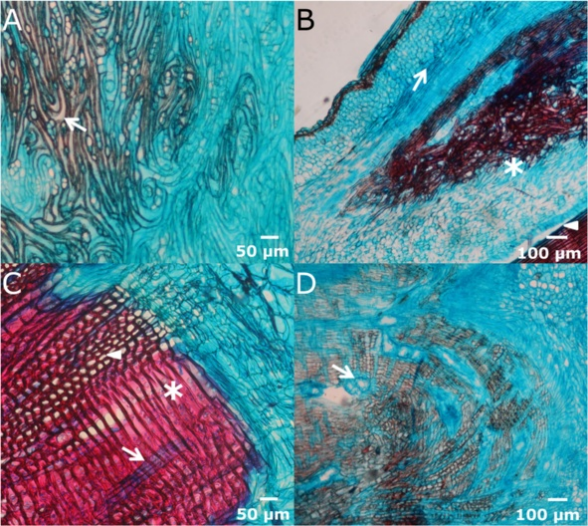
**De-orientation of tracheids. A**: Irregular and de-oriented tracheids (arrow), as seen in a tangential section of a healing upper margin **B**: Radial section of a column-like structure, showing the wound surface (arrowhead), the ventral zone with disordered tracheids and parenchymatous cells (asterisk) and normal, axially oriented xylem and phloem, (arrow), as well as the outer periderm. **C**: Series of axial tracheids (arrowhead) next to horizontally disposed tracheids (asterisk) and radius (arrow) in a cross section. **D**: Resin ducts tangentially oriented (arrows), as seen in a radial section of the upper margin.

In the irregular upper margin of girdling wounds and in the extreme of column-like structures highly crooked and disordered tracheids develop (Figure 10A). This feature can be due to the perception of surface in different directions and to the altered hormone flux, as reported by Sachs & Cohen [58] or Kurczynska & Hejnowicz [59]. This process can ultimately lead even to the differentiation of radial series of normal, axially oriented tracheids next to tangentially oriented ones. In these regions, also tangential resin ducts are developed, perpendicular both to axial and radial canals (Figure 10B-D).

Malformation and disorganization of tracheids in woundwood, as well as the later recovery of a normal pattern has been recently described in fire scars in different conifers [22] or in mechanical injuries in the stem of *Pinus sylvestris* [42] or even in overgrown stumps of felled *Pseudotsuga menziensii* [60]. This disorganized xylem, with an increased proportion of resin ducts and associated parenchyma imply an evident disadvantage for the circulation of water and nutrients through the traumatic xylem [22]. However, the fast restoration of the proper cambial polarity after reconnection allows the stem to successfully recover its hydraulic efficiency and surmount the injury. Meanwhile, if the injury is not too deep, water can be transported via the xylem of the previous years, which in conifers remain fully functional for several years, as well as through the unwounded sector of the stem.

## Conclusion

Vigorous and fast healing ability makes *P. canariensis* a suitable model species for the study of the response to wound stress in conifers. Thus, we have detected a faster response than previously reported for other gymnosperms with an initial lignification and suberization of cortical parenchymatous cells and the development of a traumatic periderm in the cortex. Conversely to angiosperms, the wound margins constitute the main regenerating focuses, through increased periclinal and radial anticlinal divisions of the cambium, while no significant proliferation from other tissues, such as phloem or radial parenchyma, has been detected.

More interestingly, this work includes the first description of column-like structures, formed after girdling, developed both from the upper healing margin and from the resin duct-associated parenchymatous cells beneath the wound surface. These structures enable fast reconnection of the vascular tissues, being subsequently embedded by the advance of the upper healing edge; additionally, they anchor the healing tissue to the wound surface.

Cambial cells perceive the relative position of the surface of the stem, which drives the orientation of additive and multiplicative divisions, and, together with altered hormone flux, leads to the formation of disordered traumatic xylem in the woundwood. While perception of verticality by plant cells (for instance, by the action of amyloplasts and/or hydrostatic gravisensing and polarly localized PIN proteins, causing geotropism in the root tip) is comparatively well known, as well as some molecular aspects of the polar differentiation of xylem and phloem (reviewed in [61,62]), the exact mechanism underlying this “horizontal” perception remains unrevealed. Although it is probably related to hormone flux and to the pressure exerted by surrounding cells, the involvement of other factors cannot be excluded. Further research is needed to clarify this point.
